# Addressing Evidence Linking Secondary Alexithymia to Aberrant Humor Processing

**DOI:** 10.1155/2019/1803624

**Published:** 2019-07-18

**Authors:** Panayiotis Patrikelis, Giuliana Lucci, Athanasia Alexoudi, Stefanos Korfias, Lambros Messinis, Grigorios Nasios, Themistoklis Papasilekas, Damianos Sakas, Stylianos Gatzonis

**Affiliations:** ^1^First Department of Neurosurgery, Medical School, National and Kapodistrian University of Athens, Greece; ^2^Department of Technologies, Communication and Society, International University of Rome “G. Marconi”, Italy; ^3^Neuropsychology Section, Departments of Neurology and Psychiatry, University Hospital of Patras and University of Patras Medical School, Greece; ^4^Department of Speech and Language Therapy, School of Health Sciences, University of Ioannina, Greece

## Abstract

In this review, we explore current literature and assess evidence linking secondary (acquired) alexithymia to aberrant humor processing, in terms of their neurobiological underpinnings. In addition, we suggest a possible common neuropathological substrate between secondary alexithymia and deficits in humor appreciation, by drawing on neurophysiologic and neuroradiological evidence, as well as on a recent and unique single-case study showing the cooccurrence of secondary alexithymia and deficit in humor appreciation. In summary, what emerges from the literature is that the cortical midline structures, in particular the medial prefrontal cortex (mPFC), the anterior cingulate cortex (ACC), and the insular cortex, seem to play a crucial role in the expression of both alexithymia and defective humor processing, while though to a lesser extent, a right hemisphere and bilateral frontoparietal contribution becomes evident. Neurobiological evidence of secondary alexithymia and aberrant humor processing points to the putative role of ACC/mPFC and the insular cortex in representing crucial processing nodes whose damage may produce both the above clinical conditions. We believe that the association of secondary alexithymia and aberrant humor processing, especially humor appreciation deficit, and their correlation with specific brain regions, mainly ACG/mPFC, as emerged from the literature, may be of some heuristic importance. Increased awareness on this topic may be of aid for neurosurgeons when accessing emotion-relevant structures, as well as for neuropsychologists to intensify their efforts to plan evidence-based neurorehabilitative interventions to alleviate the deleterious effects of such interpersonal communication deficits.

## 1. Introduction

Alexithymia, literally meaning “without words for emotions”, is used in the nosologic nomenclature to describe (1) a specific disturbance of emotional processing such as difficulties in identifying and verbalizing emotions, (2) difficulty in distinguishing between feelings and somatic sensations that accompany emotional arousal, and (3) externally oriented thinking and impaired symbolic activity [1, 2].

The prevalence of alexithymia is fairly high among patients with psychiatric disorders [3], since alexithymia may predispose to or even exacerbate preexisting psychopathology. It has been seen that alexithymic individuals may often show significantly higher levels of “functional” somatic and psychiatric symptoms such as anxiety and depression than nonalexithymics (e.g., [4]). For instance, among patients with conversion disorder, alexithymia was higher among those who attempted suicide [5]. Literature views a possible relationship between alexithymia and increased suicide risk as mediated by depressive symptoms (for a review see [6]). Moreover, in a prototypical study assessing the relationships between alexithymia, suicidal ideation, and serum lipid levels in a sample of drug-naïve adult outpatients with obsessive compulsive disorder (OCD), it has been seen that alexithymics with comorbid OCD may exhibit deregulation of the cholesterol balance, which in turn may be linked to suicidal ideation [7].

Alexithymia observed in people who have suffered from brain injury and manifesting symptoms resembling those of primary (congenital) alexithymia is referred to as “acquired alexithymia” or “secondary alexithymia.” Clinical cases of secondary alexithymia are largely neglected and not well understood [8]. What we know is that secondary alexithymia is often more resistant to treatment [9] and is not associated with a specific premorbid personality, while on the other hand it presents with cognitive impairment [10].

The lack of the sense of humor is another important aspect of alexithymia that should be further investigated, especially since the ability to produce and comprehend humor is a critical interpersonal ability in promoting suitable social interactions. Humorlessness, besides other subjective symptoms, i.e., lack of insight, emotional distance, somatizing tendency, and rigidity, are often reported by alexithymic individuals and their relatives [11], though they are not part of the core symptoms of alexithymia.

In this review, we attempt to explore current literature and evaluate evidence linking secondary alexithymia to aberrant humor processing, in terms of their neurobiological underpinnings. In addition, we suggest a possible common neuropathological substrate between secondary alexithymia and deficits in humor appreciation, by drawing on neurophysiological and neuroradiological evidence, as well as on a recent and unique single-case study showing the cooccurrence of secondary alexithymia and deficit in humor appreciation [12]. However, our main objective is to launch theoretical reflections about a putative neurobiological rapport relating secondary alexithymia and aberrant humor processing, particularly in the direction of a selective anterior cortical midline structure involvement as suggested by our prototypic case study [12], and supported to some extend by the relevant literature on secondary alexithymia, with the latter providing evidence of an also right hemisphere involvement. On the contrary, it is beyond the scope of this paper to provide a complete and detailed account of the literature and relative data. Therefore, our report focuses on the most relevant/interesting studies relating to this goal instead of considering a complete literature panorama.

## 2. Secondary Alexithymia Theories

The most relevant theories regarding the aetiology of secondary alexithymia emphasize the role of neurophysiological factors. The most prominent among them, proposed by Hoppe and Bogen [13], links alexithymia to a functional commissurotomy, which is an interhemispheric inhibition resembling the state of surgical cerebral commissurotomy sectioning the corpus callosum [14]. The authors [7] suggest that abnormal interhemispheric communication reduces coordination and integration of specialized functions, determining an abnormal state of inhibition of neural information, normally transmitted from one hemisphere to another throughout the corpus callosum.

Lane et al. [15] proposed an Anterior Cingulate Cortex (ACC) damage hypothesis linking alexithymia to ACC activity deficit during states of emotional arousal. They reported a strong correlation between emotional arousal and blood flow in the cingulate cortex of a female sample whose emotional awareness was assessed before PET imaging.

Some authors have associated alexithymic features with right hemisphere damage following stroke [16] consistently with the role of the right hemisphere in emotion processing [17, 18]. According to this alexithymia's right hemisphere activation hypothesis, the genesis of alexithymia implies a relation between the activation levels of the right hemisphere and alexithymia features, i.e., alexithymic patients could suffer a deficit in the right hemisphere emotional processing [8, 19].

By contrast, Becerra et al. [20] reported the case of a patient who developed alexithymia following a motor vehicle accident that is inconsistent with both functional commissurotomy and the ACC damage hypothesis, given that the damage of their patient was confined to the lateral, rather than medial, aspects of the cortex, in particular in the lateral frontal and parietal lobes. Moreover, the patient's damage was not confined to the right hemisphere, but rather distributed between both hemispheres, weakening the alexithymia's right hemisphere activation hypothesis.

Furthermore, a defective emotional verbal labeling hypothesis has been advanced [21], suggesting a defective emotional information transfer from the right hemisphere to the “language centre” of the left hemisphere that makes impossible labeling emotional information. Others have suggested that once the orderly linkage from body stimuli to emotions and symbolic language fails to work, a poor symbolization of bodily information and alexithymia is induced [22]. More specifically, such linkage is assumed to be mediated by the default mode network, i.e., a set of functionally interconnected brain nodes including the dorsal and ventral medial prefrontal cortex, the medial and lateral parietal cortex, the posterior cingulate cortex, and the temporal cortex [23–25].

Hobson et al. [26] suggested that impaired object-naming ability is linked to alexithymia, particularly whenever emotional identification is called upon. They also supported a large body of evidence for a putative link between verbal abilities and alexithymia, regarding both secondary alexithymia and developmental cases. Evidence of impaired interoceptive functioning in alexithymic persons supports the key role of general interoceptive impairment in giving rise to alexithymia [27, 28]. In brief, damage to areas in the inferior frontal gyrus, commonly considered to be an important language region, and in the anterior insula support the notion that alexithymia may arise from disruption in both language and interoception.

Schäfer et al. [29] reported the case of a patient who sustained a right anterior cingulate infarct. Specifically, MRI revealed a large recent infarct in the right ACC and in the adjacent corpus callosum. The lesion spread into the middle portion of the medial frontal cortex, while there was a small infarct lesion in the posterior part of the corpus callosum. The patient manifested an alexithymia-like disorder, as well as abnormal emotion processing including a right hemisphere emotional face perception deficit, as confirmed by ERP recordings.

The authors [29] hypothesized that such a lesion produced a sort of pathological interference in a critical node of a large-scale network subserving affective control of behavior. Ho and Lee [30] presented the case of a woman who, after falling to the ground during horse riding and crushing her skull, became alexithymic as a result of damage in her right primary visual cortex (Brodmann Area 18/19), suggesting that emotional dysfunctions, although frequently associated with abnormal “top-down” processing, may also be caused by damage to the “bottom-up,” more elementary sensory processes.

Finally, Freyberger [31] has proposed to consider secondary alexithymia as stress reaction. In other words, secondary alexithymia is a coping mechanism which aims at protecting the self against emotional distress associated with situations of intense vulnerability [32].

Before proceeding to the main findings of the review, we have considered as necessary to provide some additional information for the reader regarding important issues relating to alexithymia such as its rapport to psychopathy, the possibility to represent a personality trait, and humor styles, as well as a potentially shared cognitive architecture for alexithymia and humor.

### 2.1. Alexithymia and Psychopathy

Starting from the observation of Apfel and Sifneos [33] that some individuals with alexithymia had psychopathic personalities, alexithymia has been typically associated with both violence and psychopathy. Hence, although alexithymia is mainly conceptualized as a personality trait and psychopathy as personality disorder, the debate on their rapport is still ongoing. However, we need to be cautious when drawing conclusions regarding such associations. Similar manifestations may be seen in both alexithymia and psychopathy, along with emotional and interpersonal sequels, and deficits in understanding self and others [34]. Moreover, empathy, insight, and introspection are lacking in both alexithymia and psychopathy [11]. However, individuals affected by alexithymia tend to be anxious, over controlled, submissive, boring, ethically consistent, and socially conforming, while people with psychopathy tend to be anxiety-free, dominant, and nonconforming [11]. Furthermore, Lander et al. [35] observed a significant association between alexithymia and secondary psychopathy (also known as “neurotic psychopathy”), but not with primary psychopathy (i.e., inability to form emotional bonds with others and a fear insensitivity). The authors interpreted this association in a twofold way: (a) emotional disregulation linking both clinical conditions and (b) failing in control of impulsivity and anxiety [35]. However, there is evidence that an emotional regulation deficit in people with alexithymia results from a cognitive processing failure, while in psychopathic people from a failure to experience emotional arousal, anxiety, and fear in particular, even if they are able to shallowly or hollowly pantomime related feelings [36, 37].

The first description of acquired psychopathy, as suggested by Damasio [38], draws back in the classical description of Phineas Gage by Harlow in 1848, following damage to the orbitofrontal cortex (OFC). Later studies on patients with OFC damage showed a close resemblance to psychopathy, called pseudopsychopathy [39], and later to acquired sociopathy [38]. However, Hornak et al. [40] suggested that the OFC damage needs to be bilateral and rather extensive to cause changes in social behavior, while, according to Hare [41], the inability of such patients to make longer-term plans (dysexecutive problems) may be accounted for and, consequently, their manifestations do not directly correspond to psychopathic tendencies. Jurado and Junque [42] also reported two patients who presented personality changes consistent with dissociability, including delinquency, as a result of an OFC lesion. Blair and Cipollitti [43] described a patient presenting acquired sociopathy (i.e., pseudosociopathy) following trauma to the right frontal region, including the OFC, and suggested that his reduced ability to generate expectations of others' negative emotional reactions, particularly anger, may account for his sociopathic manifestations. Müller et al. [44] reported two detained sexual offenders with personality disorder and posttraumatic pseudosociopathy after frontal lobe damage. Both did not have identifiable MRI evidence of structural damage but had decreased glucose metabolism in the left frontobasal and basal ganglia regions in the former, and in the right prefrontal and right insular cortices in the latter, as suggested by PET scan.

### 2.2. Is Alexithymia a Personality Trait?

Another conflicting topic is whether alexithymia constitutes a personality trait or a distinct nosographic entity. A number of leading authors have suggested that alexithymia represents a relatively stable personality trait predisposing people to develop psychopathology, while the supposed characterologic stability becomes accentuated by the state of illness, returning to preexisting conditions after the dissipation of symptoms [45–47]. However, the stability hypothesis cannot represent a conclusive finding supporting alexithymia as a personality trait, since it cannot tell us about the premorbid levels of alexithymia in patients suffering mental illness. Due to the considerable overlap between alexithymia and anxiety and depression symptoms occurring in patients with affective disorders [48], future studies evaluating alexithymia among psychiatric populations should control for the severity of anxiety and depression before concluding that alexithymia is a personality trait predisposing to mental disorders.

### 2.3. Cognitive Psychology of Humor

At a cognitive level, the potential overlap between humor processing and alexithymia may be ascribed to processes both related to abstract thinking, mental flexibility, individual memory, and interoceptive awareness.

Although humor appreciation requires the conjunct contribution of higher level cognitive processes and affective responsiveness, the specific cognitive operations required to understand humor include abstract reasoning, cognitive flexibility, and effective memory. In order to understand humor, the ability to disregard the literal meaning and to derive a deeper-extended meaning from what is not explicitly stated is required [49]. Moreover, there is evidence for a theoretical relationship between sense of humor and cognitive flexibility [50]. Humor is a coping strategy implying positive reappraisal and processing of witty verbal expressions which allows people to distance themselves from stressful events or situations, thus gaining perspective [51]. Psychological inflexibility can be thought of as being excessively entangled in experiential avoidance and cognitive fusion. A link between alexithymia and psychological inflexibility has been hypothesized, by focusing on the latter as a sort of inability in choosing behavior in line with identified values and goals due to difficulties in connecting with the present moment fully, and blindly sticking to rigid rules [52, 53]. Additionally, cognitive flexibility may also be conceived in terms of thought styles, such as lateral thinking as opposed to vertical thinking, convergent thinking as opposed to divergent thinking, and complexity as opposed to rigidity [54].

Of importance to our discussion on the mental operations involved in humor processing is the incongruity resolution theory [55–57], based on the discrepancy between abstract ideas and real things. The resolution phase is thought to be a problem-solving operation, an effort to draw information or inferences leading to the detection of a link or a fit between the initial body of the humoristic stimulus and its ending.

### 2.4. Right Hemisphere and Individual Memory

Individual memory, meaning memory for subjective states and orientation to self and surroundings, may be disturbed following right hemisphere lesions, against a background of intact verbal and logical memory [58, 59]. This finding stemming from the classical work of Luria seems comparable to some extent with recent evidence linking right hemisphere damage to the manifestation of secondary alexithymia [16], since the right hemisphere has been extensively conceptualized as being devoted to emotional processing.

### 2.5. The Role of Interoceptive Awareness

Interoceptive awareness is also linked to emotional and social cognition. Quite recently, interoception has been conceptualized within a predictive coding framework [60]. The predictive coding framework states that resourcing to previously encode internal sensory cues is imperative for linking personal emotional state to external events. Impairments in predicting internal sensations may hinder the formation of associations between those sensations and salient social or emotional external events, possibly contributing to social and communication failures. Chronic abnormalities in interoceptive predictive coding may progressively lead to a sort of blindness for interoceptive cues, which become then habitually disregarded, and consequently giving rise to a pathological feedback loop (e.g., impaired attention makes cues less predictive, which then reinforces inattention). Such difficulties are encountered in people in the autistic spectrum, manifesting difficulty using prior sensory experience to make accurate predictions of future sensory events [61]. The neuroanatomy of interoceptive inference has been mapped onto several neuroanatomical substrates, such as the anterior insular cortex (AIC), ACC, the subgenual cortex (SGC), and OFC. Thus, interoceptive awareness represents a further cognitive aspect of secondary alexithymia, since the insular cortex is particularly involved in interoceptive processing.

### 2.6. Humor Styles

Relative to the humor styles, their original conception is ascribed to the Humor Styles Questionnaire (HSQ) measuring two main factors involved in humor: one used to enhance the self or one's relationships with others and another suggesting whether humor is relatively benevolent or potentially detrimental and destructive. The combination of these factors gives rise to four different humor styles: affiliative (jokes everyone might find funny), self-enhancing (find the humor in everyday situations as a copying strategy), aggressive (put-downs or insults targeted toward individuals), and self-defeating (an unhealthy form of humor, also implemented as a bullying avoidance strategy) [62].

## 3. Materials and Methods

A systematic narrative review was conducted in compliance with PRISMA guidelines [63]. The time period searched was 1970 to 2017 using the National Library of Medicine PubMed database (http://pubmed.gov) and Medscape.

The search keywords were “organic,” “acquired,” “primary,” “secondary,” “humor” AND “detection” OR “appreciation,” AND “styles,” AND “traumatic,” “brain,” “injury,” “psychopathy” AND “personality” AND “trait,” “psychopathy,” “memory,” “interoception,” “flexibility,” “cognitive” AND “psychological” AND “processing,” “abstract” AND “thinking” AND “reasoning,” “incongruity” AND “resolution,” “inference,” “inflexibility,” “neurological” AND “disorders,” “diagnostic” AND “classification,” “hemispheric” AND “asymmetry,” “neuroanatomy,” “neuroimaging,” “electrophysiology,” “neurophysiology.” A combined search of the above terms with the term “alexithymia” has been undertaken.

**Figure 1:**
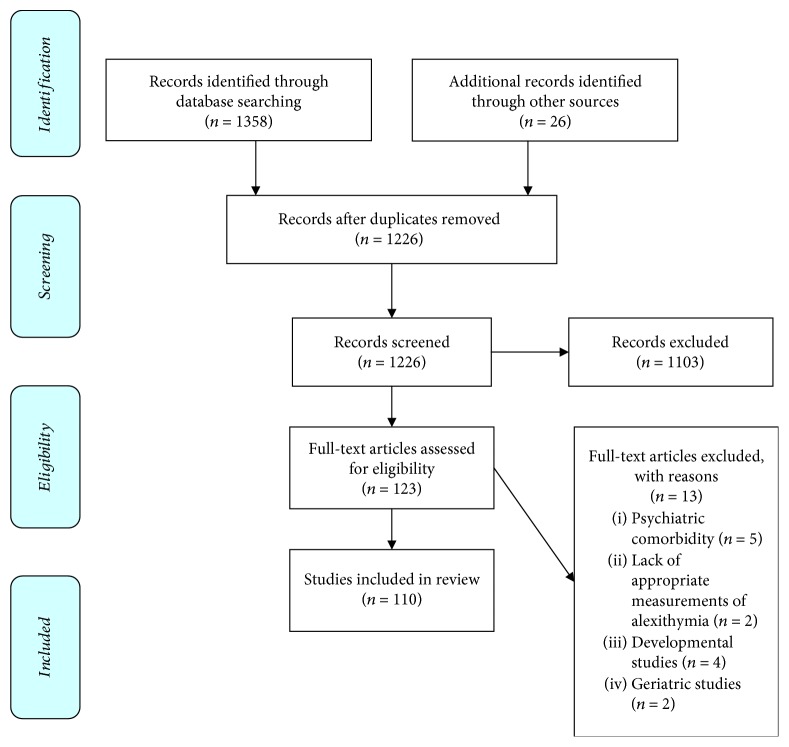
PRISMA flow diagram. The flow diagram depicts the flow of information through the different phases of a systematic review.

From the above search, 1346 articles were identified, 98 of which were included/selected for this review. The inclusion criteria for the selection were based on the preestablished inclusion criteria (see below) and studies relative to the review topic reported within the initially identified papers (Figure 1).

## 4. Inclusion Criteria

Studies needed to comply with the following criteria to be included in the review:

1. The sample consisted of adult participants only (i.e., the mean or median age of the sample was equal to or above 18 years)
2. Since secondary alexithymia is a less studied topic and as such has been investigated by fewer studies, we used neurobiological evidence deriving from both secondary and acquired cases, and occasionally, from congenital alexithymia cases. Though further research is warranted to clarify if secondary alexithymia should be considered as a separate clinical entity, as suggested by Messina et al. [10], hereby we use the expression “alexithymia” under the rubric of secondary alexithymia
3. The great majority of studies of either secondary or congenital alexithymia have implemented the 20-Item Toronto Alexithymia Scale (TAS-20) to diagnose the presence of alexithymia symptoms by administering it to patients and matched controls, or to patients only
4. In our work, only peer-reviewed journal papers written in English have been considered, except for the inclusion of three German articles (reported in the identified English language literature)
5. Neuroimaging studies were included in this review whenever they were suggested by the identified literature using the abovementioned methods and inclusion criteria

## 5. Results

### 5.1. Alexithymia Studies

#### 5.1.1. Neuroradiological and Electrophysiological Evidence

It has been assumed that a pool of anterior brain structures, i.e., PFC, ACC, frontostriatal network, insula, amygdala, corpus callosum, and anterior commissure, plays an important role in emotional processing and expression in primary alexithymia [64]. Berthoz et al. [65] sustained that alexithymia, as a deficit of higher order appraisal of emotional stimuli, may be linked to the ACC and mediofrontal cortex (mFC) malfunction, while amygdalar, hippocampal, and hypothalamic processing links to lower order aspects of emotional stimuli (i.e., the perceptual information of Reiman et al. [66]), remain intact.

Furthermore, these authors observed that the modulation of ACC and mFC activity caused by the emotional valence of stimuli was aberrant in alexithymic subjects, showing activity reduction and increase toward intense negative and positive stimuli, respectively [65]. The authors concluded that primary alexithymia reveals itself by exacerbating personality traits, and usually plays a role in affect regulation associated with circumscribed valence-dependent differences in the activity of ACC and mFC during emotional stimuli processing [65].

Lane et al. [15] proposed an ACC damage hypothesis (the blind-feel hypothesis), linking alexithymia to ACC activity deficit during states of emotional arousal. In particular, they regard alexithymia as a deficit in the conscious self-awareness of emotions and postulate that a neural disconnection mechanism prevents interoceptive information from adequately informing cortical regions, including ACC. In their positron emission tomography (PET) study, the authors reported a strong correlation between emotional arousal and the flow of blood in the anterior cingulate cortex. Evidence pointing to the same direction concerns the relation between low oxygen pressure, reflected in the low hemoglobin levels of oncologic patients, and alterations in ACC functioning [67]. Furthermore, other PET studies showed not only lower activation rates in the right orbital-PFC [68], as well as in the right frontal areas, but also higher activation rates in the left frontal cortices during recall of autobiographic emotional information [69] in alexithymic individuals as compared to those in healthy individuals.

A deregulation of emotional appraisal processes in congenital alexithymia was suggested by electrophysiological studies detecting a decrease of the upper theta band over the left frontal cortex during responses to emotional stimuli [70].

In sum, the relationship between primary alexithymia and the PFC activity has not yet been adequately addressed and it seems to be still somewhat equivocal.

#### 5.1.2. Frontostriatal and Limbic Contributions to Alexithymia

Since the striato-thalamo-cortical circuitry is likely to support emotional processing [71], various structures involved in that circuitry, such as the globus pallidus, ventral striatum, and caudate and subthalamic nuclei have been linked to both congenital [72] and secondary alexithymia [9]. There is evidence suggesting lower right caudate nucleus activation in response to angry facial stimuli even among healthy individuals with high levels of alexithymic traits [73].

A severe and resistant form of secondary alexithymia associated with bilateral globus pallidus hypoxic brain damage after carbon monoxide poisoning has been reported [9], as well as emotional awareness impairment following globus pallidus anoxic lesion [74].

The amygdala and its projections to subcortical structures, i.e., the hypothalamus, the hippocampus, and the red nucleus of the stria terminalis; the preoptic area; and the periaqueductal grey matter [75], make up a network assumed to be a phylogenetically ancient system for emotional regulation. The amygdala mediates both inborn emotional and acquired behaviors [76], while the prefrontal cortex, especially the orbital and medial portions, is related to emotional feeling which in turn links to some extent to emotional reflection [77]. If the medial PFC does not inhibit the amygdala, then the latter would trigger preprogrammed and evolutionary archaic emotional behaviors, rendering emotional feeling and reflection purposeless.

Evidence that aberrant forms of the cognitive processing of emotional information inhibit the impact of such amygdalar inputs led some authors to postulate four different alexithymic personality types within the broader concept of alexithymia [64]. These types may possibly reflect childhood's abnormal experiences, leading the main cerebral components regulating emotional experience, i.e., the amygdala and the prefrontal cortex, to remain functionally fixated at early stages of development. For instance, Patrikelis et al.'s [12] patient (see below) personality type seems compatible with Bermond et al.'s [64] second personality type, described as “…entailing a strong tendency to activate cognitive processing of emotional information modules which inhibit other neural modules involved in emotionalising…”, for which persons mainly experience emotionally detached emotion-related cognitions. Unfortunately, Bermond's typology is referred to as primary alexithymia, and it may not be applicable to secondary alexithymia cases, like the abovementioned, at least until a correlation between the alexithymic types and brain structures—or systems—is not revealed. Some authors [20] claimed that the debate on whether the symptoms of secondary alexithymia have different underlying neuronal mechanisms and clinical expressions from those of the nonsecondary one is still ongoing, though secondary alexithymia does not always result as clinically different from primary alexithymia cases. Furthermore, the difficulty in differentiating secondary from nonsecondary alexithymia is due to their shared difficulties in both identifying and verbalizing emotions, as well as the presence of stimulus-bound behavior (concrete and reality-based thinking).

#### 5.1.3. Inflammation and Hormone Imbalances

Research findings likely display a consistency with the “stress-alexithymia hypothesis” [78]. An association between alexithymia and higher circulation levels of acute phase proteins, especially C-reactive protein, has been reported by several studies (e.g., [79]). Moreover, the proinflammatory and anti-inflammatory cytokine balance of alexithymics may be tuned toward a proinflammatory imbalance with a concomitant altered cell-mediated immunity.

Recent studies have associated specific effects of this trait and its subfactors with hypothalamo-pituitary-adrenal (HPA) axis markers during stressful conditions (e.g., [80]). Accordingly, a pioneering study which addressed the putative link between alexithymia and its subfactors on HPA and sympatho-adrenal medullar (SAM) response to healthy controls showed that among all tested stress indicators, cortisol was the only variable positively related to alexithymia, probably pointing to difficulties with the identifying feeling subfactor. Alexithymia was associated with significantly increased cortisol before, during, and after the stress exposure, while no link was found between alexithymia and SAM axis [81].

#### 5.1.4. Alexithymia in Neurological Patients

Although alexithymia is likely to represent a feature of neurological disease, it is not clear how independent it is from other nosological entities, such as depression and anxiety, frequently encountered as comorbidities in neurological patients. Neurological conditions that received attention for the presence of alexithymia are traumatic brain injuries (TBIs), stroke, epilepsy, and, to a lesser degree, movement disorders and multiple sclerosis. Although there is a relatively high incidence of secondary alexithymia among people who have suffered head injuries (30-40%), the great variability of lesions in TBIs, in terms of location, severity, and extent and the often diffuse nature of axonal injury [82], as well as other methodological issues, e.g., the inability of TBI patients to identify and consequently report their own emotional state by means of self-reported questionnaires [82], make TBIs hard to study.

Williams and Wood [83] have given a possible interpretation for the presence of secondary alexithymia in TBIs suggesting an inverse relationship between alexithymia and emotional empathy. They believe that the capacity for emotional empathy may operate independently of alexithymia after TBI, possibly reflecting the impact of diffuse brain pathology on various emotional circuits, mediated by complex bidirectional and mutually inhibitory interconnections between the ventral prefrontal cortex (VPFC) and the amygdala [84, 85].

Since VPFC is highly vulnerable to the effects of acceleration-deceleration forces in TBIs [86, 87], neural networks responsible for social cognition and cognitive appraisal of stimuli separately from their somatic counterpart and depending on VPFC may be disrupted. This disruption renders patients unable to experience the emotional attributes of stimuli (e.g., Adolphs et al. [88]). Consequently, damage to the VPFC may selectively compromise the ability to recognize one's own emotions and to empathize with others' emotions.

More recently, Hogeveen et al. [89] have suggested that pronounced damage of the anterior insula induces increased levels of alexithymia, providing evidence for a critical role of this region in emotional awareness.

In the context of epilepsy research, alexithymia has been predominantly investigated in patients suffering from psychogenic nonepileptic seizures (PNES), and, to a lesser extent, in patients with epilepsy. Symptoms of psychological trauma and cynicism in patients diagnosed with PNES were associated with alexithymia. These findings are encouraging, as they assist in better understanding the condition and in treatment design for this subset of patients. A recent review [90] reports only two studies presenting alexithymia in conjunction to epilepsy and PNES. The first [91] suggests that “about 30% of both groups scored in the alexithymic range.” In contrast, alexithymia in PNES was estimated at around 90.5% and in epilepsy at 76.2% [92]. Myers et al. [90] suggest that alexithymia rates at around 36.9% of their sample with PNES and 28.6% in that with ES and are consistent with the prevalence rates of alexithymia reported in the study by Tojek et al. [91]. They [90] found a positive correlation between alexithymia and heightened symptoms of anxiety and autonomic hyperarousal, intrusive experiences, dissociation, and defensive avoidance, interpreting the above in the light of alexithymia's association with trauma, since the former represents a condition that would be expected to result from physiological and cognitive deregulations following trauma. More specifically, they found an association of alexithymia with psychological trauma and cynicism in PNES patients.

Moreover, few studies taking alexithymia into account have been conducted in the domains of multiple sclerosis and movement disorders [93].

### 5.2. Humor Processing Studies

#### 5.2.1. Neuroradiological Evidence

The ability to produce and comprehend humor is a critical interpersonal ability in promoting suitable social interactions. Humor processing entails many cognitive aspects, such as (a) the setup of the joke—associated with temporal lobe (i.e., temporal pole and anterior superior temporal sulcus (aSTS)) activation [94–97]; (b) humor comprehension and detection/resolution of incongruity—involving the inferior frontal gyrus (IFG) [97–100] and regions around the temporal parietal junction (TPJ) [94, 97, 101]; and (c) the attribution of mental states to the characters of the joke related to the activation of the anterior medial prefrontal cortex (amPFC) [102, 103]. Moreover, it could be claimed that affective components of humor processing are related to the medial ventral prefrontal cortex (mvPFC), subcortical nuclei (nucleus accumbens), bilateral insular cortex, and left amygdala functioning [94, 96, 98, 99, 104].

Some studies emphasized cerebral activations in regard to structural elements of humoristic stimuli. Specifically, phonological puns activate areas involved in sound processing, i.e., the left inferior precentral gyrus and insula, whereas semantic jokes activate regions processing word meaning, i.e., the left posterior inferior temporal gyrus (pITG) and the right posterior middle temporal gyrus (pMTG) [98]. Finally, some differences were observed comparing visual-based vs. language-based humor. In brief, the former activates, among others, the bilateral higher-order visual cortex, TPJ, middle frontal gyrus (MFG), and precuneus, while the latter specifically activates the MTG, IFG, and ITG [100].

According to Moran et al. [96], the neural underpinnings of humor detection and appreciation are functionally separable, with the posterior temporal and inferior frontal regions devoted to resolving contextual ambiguities and recruited during humor detection, and the insular cortex implicated in visceral responses characterizing the mirth experience.

### 5.3. Joint Evidence for Alexithymia and Defective Humor Processing

In a unique behavioral genetic study from Atkinson et al. [105] on the rapport between congenital alexithymia and humor styles, it has been found that positive humor styles correlated negatively with alexithymia, while maladaptive humor styles (self-defeating and aggressive humor) correlated positively with alexithymia. Phenotypic correlations between alexithymia and humor styles were primarily attributable to associated genetic factors and, to a lesser extent, to nonshared environmental factors. A possible reason accounting for correlations between humor styles and alexithymia in the above study is that both correlate with empathy. Affiliative humor (i.e., positively involving others and enhancing social interaction) and self-enhancing humor (i.e., enhancing the self) show a significantly positive correlation with empathy [106] that in turn is negatively correlated with alexithymia [107]. Moreover, a negative correlation between aggressive humor and empathy has been reported [106]. With regards to the neural underpinnings of empathy, it has been observed that perceiving and assessing painful situations experienced by others induce significant bilateral changes in the anterior cingulate, the anterior insula, the cerebellum, and, to a lesser extent, the thalamus, structures known for playing a significant role in pain processing [108].

A recent case study described a patient who was operated for a huge frontal-groove meningioma that presented with secondary alexithymia and impaired humor appreciation as the result of damage to the ventral-rostral portions of the anterior cingulate gyrus/mPFC, which prevented the patient from assessing the salience of emotion and motivational information and generating emotional reactions; consequently, he had difficulties in experiencing emotions, knowing how he and others feel, and enjoying humor [12] (Table 1).

However, evidence on secondary alexithymia and its relation with humor deficits is scarce and even absent.

## 6. Discussion

By drawing upon evidence from current literature and expert opinion derived from clinical practice, we suggest that clinicians and researchers working in the area of neurology, psychiatry, and clinical neurosciences should be aware of the putative link between secondary alexithymia and abnormal humor processing. Increased awareness on this topic may aid neurosurgeons when accessing emotion-relevant structures, as well as clinical neuropsychologists to plan evidence-based neurorehabilitative interventions in order to alleviate the deleterious effects of such interpersonal communication deficits.

Secondary alexithymia may be viewed as a specific variant of secondary alexithymia resulting from damage in specific brain regions including the ACC, basal ganglia, insula, right hemisphere, and corpus callosum [10], particularly those mediating emotional awareness or linking somatic signals to their abstract linguistic-symbolic counterparts. However, to our knowledge, the study of a possible common neurological link between alexithymia and aberrant humor processing has not yet been systematically addressed, and has been limited mainly to single-case study observations [12] or even to anecdotal evidence.

On the other hand, humor detection and appreciation are dissociable processes from both functional and structural points of view, with the posterior temporal and inferior frontal regions recruited during humor detection in order to resolve contextual ambiguities and the insula implicated in visceral responses characterizing the mirth experience [96]. Furthermore, limbic regions involved in generating emotional responses (i.e., amygdala, nucleus accumbens, hypothalamus, and anterior insula) are regulated by the ventral ACC/mPFC. The amygdala mediates both inborn emotional and acquired behaviors [76], while the PFC, specifically its orbital and medial portions, is related to emotional feeling which in turn encompasses to some extent emotional reflection. Disconnections between the mPFC and the above limbic components may thus produce a state of humor appreciation deficit, a sort of humor anhedonia.

In summary, what emerges from the literature is that the cortical midline structures, in particular the mPFC, ACC, and insular cortex, seem to play a crucial role in the expression of both alexithymia and defective humor processing, while to a lesser extent, a right hemisphere and bilateral frontoparietal contribution becomes evident. Consistently, a recent theory assumes that neural activity in the anterior cortical midline structures is critical for emotional feeling [109], while focal node dysfunction within the emotional processing networks may lead to selective patterns of neurobehavioral impairments, often leading to a more generalized network malfunction. Taken together, neurobiological evidence of secondary alexithymia and aberrant humor processing points to the putative role of ACC/mPFC and the insula in representing crucial processing nodes whose damage may produce both the above clinical conditions. We believe that the association of secondary alexithymia and aberrant humor processing, especially humor appreciation deficit, and their association with specific brain regions, mainly ACG/mPFC and the insular lobe, as emerged from the literature, may be of some heuristic importance.

We speculate that the above neuroanatomical findings linked to the expression of secondary alexithymia may be conceptualized as a possible breakdown of a network subserving integration of interoceptive, affective, and linguistic information. In terms of the Lurian model of brain function, the emergent lesional pattern (in particular, ACC/mPFC and the insula) leads us to hypothesize a possible disruption of the functional interplay between the first and the second functional blocks, as suggested by Luria's functional brain model [110]. The first block (in our case ACG/mPFC) is mediated by the reticular formation and the medial surface of the cerebral hemispheres, while the second (in our case insula) relies on the association cortices. In the light of this model, we assume that a disruption could occur at any level of information processing, normally taking place due to the cooperation of the above functional blocks.

This is quite crucial in neurosurgery, when the surgeon has to deal with brain pathologies related mainly with ACC and mPFC, such as brain tumors (meningiomas, gliomas), trauma (cerebral contusions), and stroke (infarctions, hemorrhages). Patients may present signs of secondary alexithymia and aberrant humor processing even before the operation, and consequently presurgical planning should include detailed neuropsychological assessment and functional neuroimaging. Intraoperatively, the neurosurgeon should protect the ACC and mPFC areas, as far as possible, in order to minimise further parenchymal damage of eloquent brain areas and consequently to avoid postoperative neuropsychological deterioration, which will make the patient's postoperative neurorehabilitation and therapeutic outcome more complex.

Moreover, increased awareness of the frequent coexistence between secondary alexithymia and humor appreciation deficit, and most importantly their shared neurobiological and cognitive underpinnings may provide a direct aid in the context of neuropsychological rehabilitation. Neurobiological (e.g., MRI and PET scans) and neurocognitive (e.g., patterns of cognitive dysfunction pointing to mental flexibility and abstract reasoning problems) evidence seen in the light of our review results may be informative with respect to the breakdown of affected neural tissues and cognitive processes and their coproducts, and consequently determine which of them should be targeted in the course of the neurorehabilitation program. In particular, mental flexibility, abstract verbal reasoning, interoceptive awareness, and right hemisphere function should be taken into consideration whenever dealing with such patients.

Psychiatrists may target neurotransmission systems linked to the emotionally relevant structures playing a decisive role in both the expression of secondary alexithymia and humor appreciation, by drawing on the anatomical distribution of abnormal imaging findings and the neurocognitive architecture of such neurobehavioral breakdowns.

Future studies should target the rapport of secondary alexithymia with humor processing by focusing on the analysis of lesions' anatomical site, extent and severity, and underlying pathophysiology, as well as patients' cognitive and psychosocial parameters. Once such issues are resolved, clinicians in the context of neuropsychological rehabilitation will be able to precisely map cognitive and affective impairments (e.g., humor appreciation deficit) and their dynamic interactions, and consequently plan effective neurorehabilitative programs to address the kaleidoscopic nature of problems in secondary alexithymia.

## 7. Conclusion

It appears that a selective involvement of the anterior cortical midline structures (mFC, ACC, and the insula, in particular) is likely to reflect the pathophysiological underpinnings of both secondary alexithymia and humor appreciation deficit. From a cognitive point of view, abstract thinking, mental flexibility, individual memory, and interoceptive awareness are the likely candidates for cognitive rehabilitation interventions in patients suffering secondary alexithymia and/or humor appreciation deficit. Finally, we hypothesize that functional breakdowns in integrating interoceptive, affective, and linguistic information may be responsible for the expression of secondary alexithymia and humor appreciation deficit, while the disruption could occur at any level of information processing, normally taking place within the domain of the Lurian first and second functional blocks.

## Figures and Tables

**Table 1:** Secondary alexithymia and humor studies.

Organic alexithymia		Aberrant humor	
Studies	Brain region	Brain region	Studies
Paradiso et al., [74] Messina et al., [67] Patrikelis et al., [12] Hobson et al., [26] Schäfer et al., [29]	Ventral-rostral ACC/*mPFC*	*IFG*, *MFG*, ITG MTG, TPJ, and precuneus	Watson et al., [100]
		*IFG*	Wild et al., [97] Goel and Dolan, [98] Bartolo et al., [99] Watson et al., [100]
		*mvPFC*	Mobbs et al., [94] Moran et al., [96] Goel and Dolan, [98] Bartolo et al., [99] Sieboerger et al., [104]
		*amPFC*	Gallagher et al., [102] Marjoram et al., [103]

Hogeveen et al., [89]	*Anterior insula*	*Insula*	Moran et al., [96]
		*Insula*, left IPG, left pITG, and right pMTG	Goel and Dolan, [98]

Hoppe and Bogen, [13] Galin, [14]	*Corpus callosum*		

Huang et al., [9] Paradiso et al., [74]	*Globus pallidus*		

Spalletta et al., [16] Ho and Lee [30] Schäfer et al., [29]	*Right hemisphere*		

Williams et al., [83]	*Bilateral frontal and parietal lobes*	Insula, *left IPG*, *left pITG*, and *right pMTG*	Goel and Dolan, [98]
